# Development of a clinical prediction model for poor treatment outcomes in the intensive phase in patients with initial treatment of pulmonary tuberculosis

**DOI:** 10.3389/fmed.2025.1472295

**Published:** 2025-03-26

**Authors:** Bin Lu, Yunzhen Shi, Mengqi Wang, Chenyuan Jin, Chenxin Liu, Xinling Pan, Xiang Chen

**Affiliations:** ^1^Department of Infectious Diseases, Affiliated Dongyang Hospital of Wenzhou Medical University, Dongyang, Zhejiang, China; ^2^Department of Neurology, Affiliated Dongyang Hospital of Wenzhou Medical University, Dongyang, Zhejiang, China; ^3^Department of Biomedical Sciences Laboratory, Affiliated Dongyang Hospital of Wenzhou Medical University, Dongyang, Zhejiang, China

**Keywords:** pulmonary tuberculosis, pulmonary CT, intensive phase, poor treatment outcome, prediction model, machine learning model

## Abstract

**Background:**

A prediction model is hereby developed to identify poor treatment outcomes during the intensive phase in patients with initial treatment of pulmonary tuberculosis (TB).

**Methods:**

The data of inpatients with pulmonary TB were collected from a tertiary hospital located in Southeastern China from July 2019 to December 2023. The included patients were divided into the modeling group and the validation group. The outcome indicator was based on a comparison of pulmonary CT findings before and after the two-month intensive phase of anti-TB treatment. In the modeling group, the independent risk factors of pulmonary TB patients were obtained through logistic regression analysis and then a prediction model was established. The discriminative ability (the area under the curve of the receiver operating characteristic, AUC), its calibration (GiViTI calibration chart), and its clinical applicability (decision curve analysis, DCA) were respectively evaluated. In addition, the prediction effectiveness was compared with that of the machine learning model.

**Results:**

A total of 1,625 patients were included in this study, and 343 patients had poor treatment outcomes in the intensive phase of anti-TB treatment. Logistic regression analysis identified several independent risk factors for poor treatment outcomes, including diabetes, cavities in the lungs, tracheobronchial TB, increased C-reactive protein, and decreased hemoglobin. The AUC values were 0.815 for the modeling group and 0.851 for the validation group. In the machine learning models, the AUC values of the random forest model and the integrated model were 0.821 and 0.835, respectively.

**Conclusion:**

The prediction model established in this study presents good performance in predicting poor treatment outcomes during the intensive phase in patients with pulmonary TB.

## 1 Introduction

In 2022, there were 10.6 million new tuberculosis (TB) cases worldwide, with an incidence rate of 133 per 100,000 people. China ranks third among the 30 high-burden TB countries, accounting for 7.1% of the global incidence. At present, the success rate of initial treatment of pulmonary TB is about 85% (1). Pulmonary TB treatment can be divided into the intensive phase and the continuous phase. Treatment interruptions frequently occur during the intensive phase (2, 3). Additionally, most adverse treatment outcomes in pulmonary TB inpatients also occur during this phase (4–6). Therefore, the intensive phase of treatment is critical for the final prognosis for pulmonary TB patients, resulting into the urgent requirement of earlier detection of treatment outcomes.

At present, the treatment effect of TB patients can be evaluated if they have turned negative on sputum smear and culture results at the end of intensive treatment phase. However, the above methods cannot be used as indicators for assessing the treatment effect of patients with bacillus-negative pulmonary TB in clinical practice (7). Additionally, for some patients with smear-positive pulmonary TB, false-negative results may exist in sputum smear for acid-fast bacilli at the end of the intensive treatment phase. This can occur due to quality problems with the randomly inspected sputum (8). Therefore, it is impossible to comprehensively evaluate the effect in the intensive treatment phase only via the sputum smear. Bacterial culture has the disadvantage of a long incubation time (negative results in liquid culture require more than 42 days) and may not yield results in cases of culture contamination (9). Consequently, the effectiveness of anti-tuberculosis treatment monitoring through direct pathogenetic testing remains challenging.

On the other hand, pulmonary imaging is fast and feasible to visualize the treatment effect. It can be used in the diagnosis and follow-up of pulmonary TB, especially in the continuous monitoring of the prognosis of patients with bacterial-negative pulmonary TB (10). Pulmonary imaging examination mainly includes chest X-ray (CXR) and lung computed tomography (CT). CXR is helpful for the rapid and cost-effective early diagnosis of TB, but using CXR to monitor the prognosis of TB is difficult (11). Because of its high resolution, pulmonary CT is significantly better than CXR in observing the absorption of pulmonary lesions during the entire treatment process of pulmonary TB. It is a highly sensitive tool for tracking the treatment efficacy among pulmonary TB patients (12), especially in the early intensive treatment phase (13).

Few prediction models use pulmonary CT as an outcome indicator to predict the effect of early anti-TB treatment in the intensive phase. Nijiati et al. developed several machine learning prediction models, but the prediction efficiency is not adequate based on the area under the curve of the receiver operating characteristic (AUC) values (13, 14). Moreover, there are fewer clinical indicators included in these prospectives studies, and machine-learning models show deficiencies in the clinical practice (15).

Thereby, it is essential to quickly screen pulmonary TB patients who have poor treatment outcomes during the intensive phase. The changes of pulmonary CT results before and after the intensive treatment phase were adopted in this study as the outcome indicators. Thus, developing such a model enables clinicians to find pulmonary TB patients with poor treatment efficacy as early as possible, enabling timely intervention.

## 2 Materials and methods

### 2.1 Inclusion of patients

Data from all inpatients in this study were extracted from the medical record information mining database of the affiliated Dongyang Hospital of Wenzhou Medical University. This database was constructed with the technical support of Le 9 Health Science and Technology Co. Ltd. All personal identification information was removed from the medical records.

*Inclusion criteria*: 1. Patients clinically diagnosed with pulmonary tuberculosis according to the Chinese Diagnostic Criteria for Pulmonary Tuberculosis (WS 288-2017). 2. The patients receiving anti-TB treatment using isoniazid, rifampicin, pyrazinamide and ethambutol (HRZE) for a two-month intensive phase.

*Exclusion criteria*: 1. Patients under 18 years old; 2. Patients with incomplete data; 3. Pregnant patients; 4. Patients with AIDS; 5. Patients who did not complete the intensive treatment phase (i.e., anti-TB treatment duration less than two months).

According to the above inclusion and exclusion criteria, patients who were first diagnosed with pulmonary TB in our hospital between July 2019 and December 2023 were finally included in this retrospective study, and all variables involved in the prediction model were collected at the time of the patients’ initial admission to the hospital.

### 2.2 Research variables

The variables included gender, age and the levels of the following indicators in the first examination after admission: creatinine, C-reactive protein (CRP), white blood cells, hemoglobin, platelet, total bilirubin, albumin, alanine aminotransferase, aspartate aminotransferase, total cholesterol, triglyceride, high-density lipoprotein cholesterol (HDL) and low-density lipoprotein cholesterol (LDL). Moreover, the information on smoking history, alcohol consumption history, medical history of diabetes, tumor, hypertension, liver disease and chronic obstructive pulmonary disease were enrolled. The presences of pulmonary cavity or tracheobronchial tuberculosis (TBTB) were identified in pulmonary CT images. The primary outcome indicator was the treatment efficacy by pulmonary CT examination after two months of intensive anti-TB therapy. If the pulmonary CT shows an increase in lung lesions compared to the pre-treatment examination results, it indicates the poor treatment effect.

### 2.3 Establishment and evaluation of prediction models

The statistical analysis in this study was done using R (version 4.2.2). The continuous variables conforming to the normal distribution were expressed as Mean ± SD and analyzed with Student’s t-test. The continuous variables conforming to non-normal distribution were expressed as the median and quartile ranges and analyzed by Mann–Whitney U-test. The categorical variable was expressed in number (percentage) and analyzed by chi square test. *p* < 0.05 indicated statistical significance.

The enrolled patients were divided into the modeling group and validation group at a ratio of 7:3 using “createDataPartition” function in the “caret” package. The “twogrps” function in the “CBCgrps” package was employed to detect significant differences of baseline characteristics between the modeling group and the validation group. Univariate analysis was conducted in the modeling group to screen the risk factors associated with poor treatment outcomes. The “boxTidwell” function in the “car” package was adopted to determine whether the continuous variables were linearly associated using logitp (*p* > 0.05). The “VIF” function was used for multicollinearity test. If the value of variance inflation factors (VIFs) was less than 5, no significant collinearity was considered. Regarding variables meeting the requirements of linear relation to logitP and no multicollinearity between included variables, a multivariable logistic regression was conducted to obtain independent risk factors for modeling. Finally, the “regplot” package was utilized to draw a nomogram to display the model. The model was evaluated from three aspects: discrimination, calibration and clinical applicability. The discrimination ability of the prediction model refers to its ability to effectively distinguish the poor treatment outcomes of pulmonary TB patients in the intensive phase, which is evaluated by AUC. Higher AUC values indicate better discrimination ability of the model. The calibration of this model was performed using the calibration chart, and the high degree of overlap between the fitting curve and the standard curve indicated the high goodness of fit. The DCA curve was applied to evaluate the clinical applicability of the model. The farther the established DCA curve is away from the two extreme curves (All curve and None curve), the better clinical applicability it indicates.

Finally, the logistic regression model established in this study was compared with the machine learning model. In the machine learning model, the methods of random forest (“randomForest” package), support vector machine (SVM, “kernlab” package), extreme gradient boosting (Xgboost, “xgboost” package) and decision tree (“rpart” package) were set by default parameters to build models. Subsequently, these four machine learning methods were integrated through stacking to establish the ensemble model (16–18), and the DeLong test was used to compare the discrimination ability between the logistic regression model and different machine learning models. A *p* value less than 0.05 indicated a significant difference in the comparison.

## 3 Results

### 3.1 Basic information of included patients

**Figure 1:**
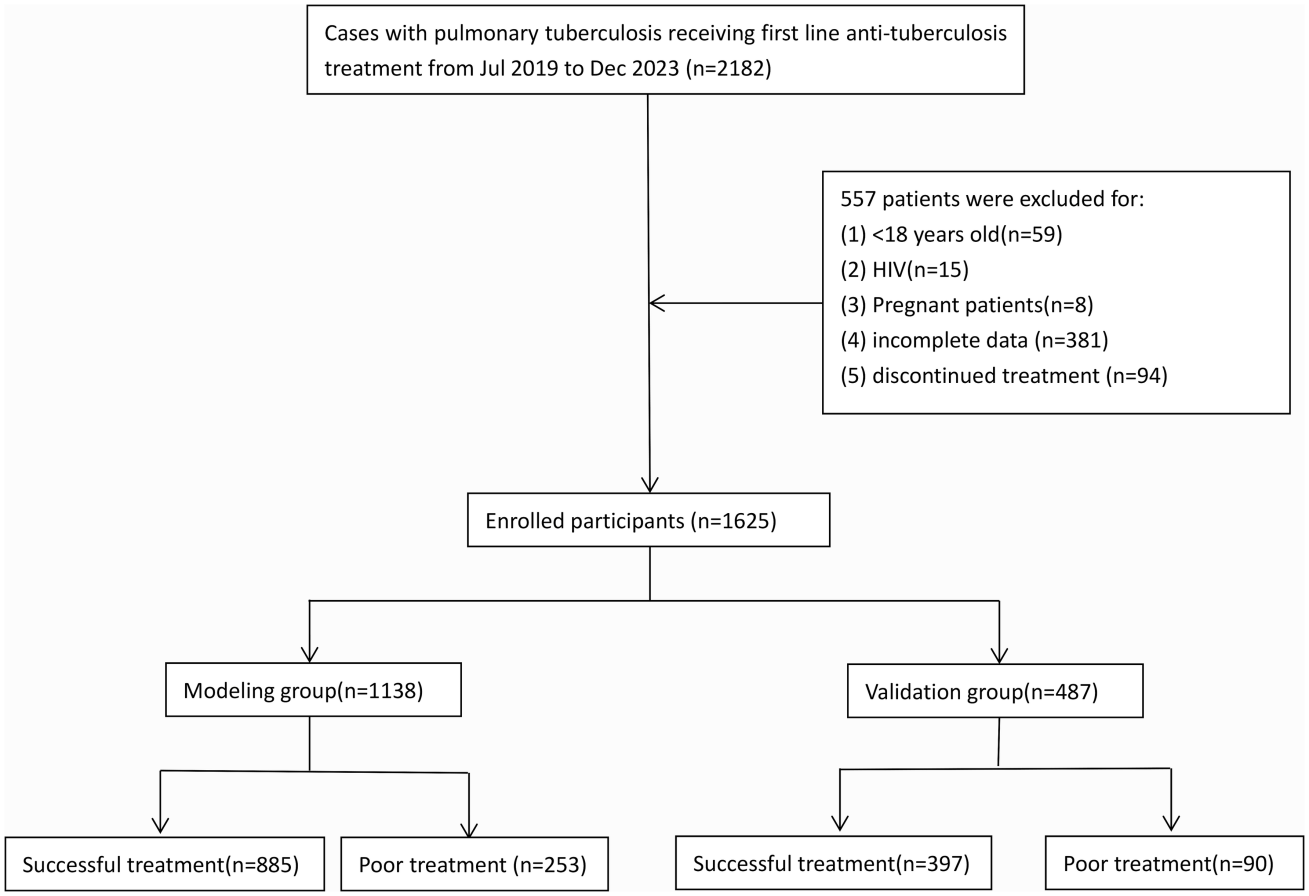
Flowchart for patient selection.

During the period from July 2019 to December 2023, a total of 2,182 inpatients with pulmonary TB received first line anti-TB protocol. Out of these patients, 557 were excluded, including 59 patients younger than 18 years old, 15 HIV patients, 8 pregnant patients, 381 patients with incomplete data, and 94 patients who did not finalize treatment. Finally, 1,625 patients were included in the study (Figure 1). Among the subjects included, 343 patients (21%) had poor treatment outcomes in the intensive phase. There were 1,138 cases in the modeling group (253 cases with poor treatment outcome) and 487 cases in the validation group (90 cases with poor treatment outcome). No significant difference in baseline characteristics between the two groups was detected (*p* > 0.05, Table 1). In the modeling group, the univariate analysis showed that the ten variables (CRP, white blood cells, hemoglobin, platelet, albumin, HDL, diabetes, tumor, pulmonary disease, and TBTB) were correlated with poor prognosis (*p* < 0.05, Table 2). The included variables were linear to the logitP (*p* > 0.05, Supplementary Table S1). No multicollinearity existed as the VIF values of all variables were less than 5 (Supplementary Table S2).

**Table 1:** Baseline characteristics of the development group and validation group^a^.

Variables	Total (*n* = 1,625)	Model (*n* = 1,138)	Validation (*n* = 487)	*p*
Gender, *n* (%)				0.064
Female	553 (34)	404 (36)	149 (31)	
Male	1,072 (66)	734 (64)	338 (69)	
Age (years)	61 (39, 74)	61 (38, 75)	61 (40, 73)	0.733
Creatinine (μmol/L)	62 (53, 74)	62 (53, 73.75)	63 (53, 74)	0.282
C-reactive protein (mg/L)	12.3 (2.2, 50.6)	12.41 (2.4, 52.25)	11.4 (1.73, 46.94)	0.156
White blood cells (10*9/L)	6.33 (5.05, 8.14)	6.29 (5.1, 8.14)	6.39 (4.99, 8.13)	0.812
Hemoglobin (g/L)	126 (111, 139)	125 (111, 139)	127 (109, 140.5)	0.531
Platelet (10*9/L)	245 (194, 304)	247 (195, 302)	241 (192, 306.5)	0.440
Total bilirubin (μmol/L)	9.6 (7.2, 12.9)	9.4 (7.1, 13)	9.7 (7.3, 12.6)	0.416
Albumin (g/L)	36.2 (31.7, 40.1)	36.1 (31.7, 40)	36.4 (31.7, 40.2)	0.769
Alanine aminotransferase (U/L)	15 (10, 23)	14 (10, 23)	15 (10, 22.5)	0.734
Aspartate transaminase (U/L)	19 (16, 25)	19 (16, 26)	19 (15, 24)	0.204
Total cholesterol (mmol/L)	3.83 (3.27, 4.48)	3.83 (3.27, 4.48)	3.84 (3.28, 4.49)	0.984
Triglyceride (mmol/L)	1 (0.76, 1.38)	1 (0.76, 1.38)	1.01 (0.77, 1.38)	0.334
HDL (mmol/L)	1 (0.81, 1.21)	1 (0.82, 1.22)	0.99 (0.81, 1.19)	0.332
LDL (mmol/L)	2.26 (1.77, 2.79)	2.25 (1.77, 2.81)	2.27 (1.79, 2.78)	0.940
Smoking, *n* (%)				0.057
No	1,016 (63)	729 (64)	287 (59)	
Yes	609 (37)	409 (36)	200 (41)	
Alcohol consumption, *n* (%)				0.529
No	1,328 (82)	935 (82)	393 (81)	
Yes	297 (18)	203 (18)	94 (19)	
Diabetes, *n* (%)				0.594
No	1,327 (82)	925 (81)	402 (83)	
Yes	298 (18)	213 (19)	85 (17)	
Tumor, *n* (%)				0.620
No	1,389 (85)	969 (85)	420 (86)	
Yes	236 (15)	169 (15)	67 (14)	
Hypertension, *n* (%)				0.070
No	1,211 (75)	833 (73)	378 (78)	
Yes	414 (25)	305 (27)	109 (22)	
Liver disease, *n* (%)				0.346
No	1,332 (82)	940 (83)	392 (80)	
Yes	293 (18)	198 (17)	95 (20)	
COPD, *n* (%)				0.829
No	1,444 (89)	1,013 (89)	431 (89)	
Yes	181 (11)	125 (11)	56 (11)	
Pulmonary cavity, *n* (%)				0.380
No	1,236 (76)	873 (77)	363 (75)	
Yes	389 (24)	265 (23)	124 (25)	
TBTB, *n* (%)				0.391
No	1,278 (79)	888 (78)	390 (80)	
Yes	347 (21)	250 (22)	97 (20)	
Smear positive, *n* (%)				0.702
No	1,132 (70)	789 (69)	343 (70)	
Yes	493 (30)	349 (31)	144 (30)	
Poor treatment, *n* (%)				0.103
No	1282 (79)	885 (78)	397 (82)	
Yes	343 (21)	253 (22)	90 (18)	

HDL, High-density lipoprotein cholesterol; LDL, Low-density lipoprotein cholesterol; COPD, Chronic obstructive pulmonary disease. TBTB, Tracheobronchial tuberculosis.

**Table 2:** Univariate analysis between successful treatment and unsuccessful treatment in modeling group^a^.

Variables	Total (*n* = 1,138)	Successful treatment (*n* = 885)	Unsuccessful treatment (*n* = 253)	*p*
Gender, *n* (%)				0.621
Female	404 (36)	318 (36)	86 (34)	
Male	734 (64)	567 (64)	167 (66)	
Age (years)	61 (38, 75)	61 (37, 75)	64 (41, 74)	0.409
Creatinine (μmol/L)	62 (53, 73.75)	62 (53, 73)	61 (51, 74)	0.209
C-reactive protein (mg/L)	12.41 (2.4, 52.25)	8.51 (1.8, 34.5)	73.83 (15.7, 110.6)	< 0.001
White blood cells (10*9/L)	6.29 (5.1, 8.14)	6.2 (5.05, 7.9)	6.82 (5.29, 8.84)	0.004
Hemoglobin (g/L)	124.48 ± 20.11	127.53 ± 19.1	113.81 ± 19.95	< 0.001
Platelet (10*9/L)	247 (195, 302)	244 (195, 296)	261 (202, 327)	0.020
Total bilirubin (μmol/L)	9.4 (7.1, 13)	9.6 (7.2, 13)	8.9 (6.8, 12.8)	0.241
Albumin (g/L)	36.1 (31.7, 40)	37.3 (33.6, 40.5)	30.9 (24.9, 36.6)	< 0.001
Alanine aminotransferase (U/L)	14 (10, 23)	14 (10, 24)	15 (10, 22)	0.804
Aspartate transaminase (U/L)	19 (16, 26)	19 (16, 25)	20 (15, 27)	0.939
Total cholesterol (mmol/L)	3.83 (3.27, 4.48)	3.85 (3.33, 4.48)	3.79 (3.09, 4.48)	0.078
Triglyceride (mmol/L)	1 (0.76, 1.38)	1 (0.75, 1.4)	1.01 (0.76, 1.33)	0.973
HDL (mmol/L)	1 (0.82, 1.22)	1.03 (0.83, 1.24)	0.92 (0.76, 1.14)	< 0.001
LDL (mmol/L)	2.25 (1.77, 2.81)	2.26 (1.79, 2.81)	2.22 (1.69, 2.79)	0.254
Smoking, *n* (%)				0.596
No	729 (64)	571 (65)	158 (62)	
Yes	409 (36)	314 (35)	95 (38)	
Alcohol consumption, *n* (%)				0.236
No	935 (82)	734 (83)	201 (79)	
Yes	203 (18)	151 (17)	52 (21)	
Diabetes, *n* (%)				< 0.001
No	925 (81)	771 (87)	154 (61)	
Yes	213 (19)	114 (13)	99 (39)	
Tumor, *n* (%)				0.017
No	969 (85)	766 (87)	203 (80)	
Yes	169 (15)	119 (13)	50 (20)	
Hypertension, *n* (%)				1
No	833 (73)	648 (73)	185 (73)	
Yes	305 (27)	237 (27)	68 (27)	
Liver disease, *n* (%)				0.781
No	940 (83)	733 (83)	207 (82)	
Yes	198 (17)	152 (17)	46 (18)	
COPD, *n* (%)				0.602
No	1,013 (89)	785 (89)	228 (90)	
Yes	125 (11)	100 (11)	25 (10)	
Pulmonary cavity, *n* (%)				< 0.001
No	873 (77)	747 (84)	126 (50)	
Yes	265 (23)	138 (16)	127 (50)	
TBTB, *n* (%)				< 0.001
No	888 (78)	742 (84)	146 (58)	
Yes	250 (22)	143 (16)	107 (42)	
Smear positive, *n* (%)				0.888
No	789 (69)	615 (69)	174 (69)	
Yes	349 (31)	270 (31)	79 (31)	

HDL, High-density lipoprotein cholesterol; LDL, Low-density lipoprotein cholesterol; COPD, Chronic obstructive pulmonary disease. TBTB, Tracheobronchial tuberculosis.

### 3.2 Variable screening and establishment of logistic regression model

The final logistic regression analysis results demonstrated increased CRP (OR 1.014), declined hemoglobin (OR 0.979), having diabetes mellitus (OR 2.159), having pulmonary cavity (OR 2.707), and having TBTB (OR 2.628) were independent risk factors related to poor treatment outcomes in the intensive phase of pulmonary TB treatment (Table 3), and were included in the model.

**Table 3:** Logistic regression analysis of independent risk factors for unsuccessful treatment in patients with pulmonary tuberculosis.

Variables	OR (95% CI)	*p*
C-reactive protein (mg/L)	1.014 (1.009, 1.018)	<0.001
Hemoglobin (g/L)	0.979 (0.970, 0.989)	<0.001
Diabetes	2.159 (1.433, 3.236)	<0.001
Pulmonary cavity	2.707 (1.840, 3.970)	<0.001
Tracheobronchial tuberculosis	2.628 (1.810, 3.808)	<0.001

OR, odds ratio; CI, confidence interval.

**Figure 2:**
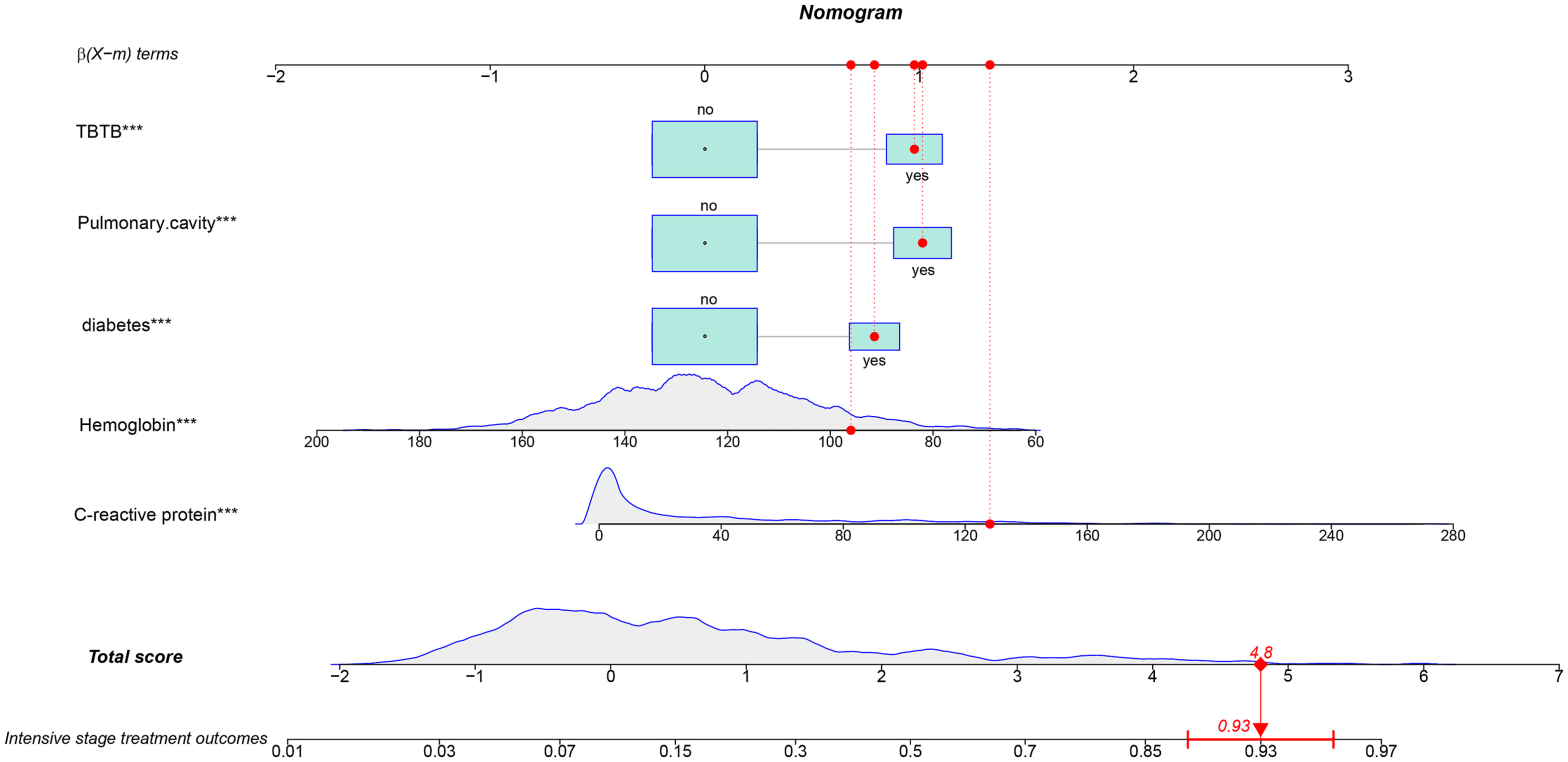
The nomogram of established model for predicting poor treatment risk in pulmonary tuberculosis patients during hospitalization. The enrolled variables were collected for the first time after admission. A patient was displayed as an example, with detailed enrolled variables labelled by red dots. The variables labelled of asterisk indicated significance in the model, *, *p* < 0.05, **, *p* < 0.01, ***, *p* < 0.001.

This model was visualized as a personalized nomogram (Figure 2). To use the nomogram, a vertical line was drawn from each variable upwards to the top scoring line and the corresponding points were recorded. Then the scores of the corresponding points of each variable were summed up to calculate the total score, based on which the prediction probability of corresponding poor treatment outcomes at the bottom of the nomogram was finally obtained. For example, the variables of one pulmonary TB patient at admission were as follows: CRP 128.1 (mg/L), hemoglobin 96 (g/L), accompanied by diabetes, lung cavities and TBTB. The patient’s total score was 4.8, and the corresponding predicted probability of poor treatment outcome was 0.93 (Figure 2).

### 3.3 Evaluation on the prediction models in the modeling and validation groups

**Figure 3:**
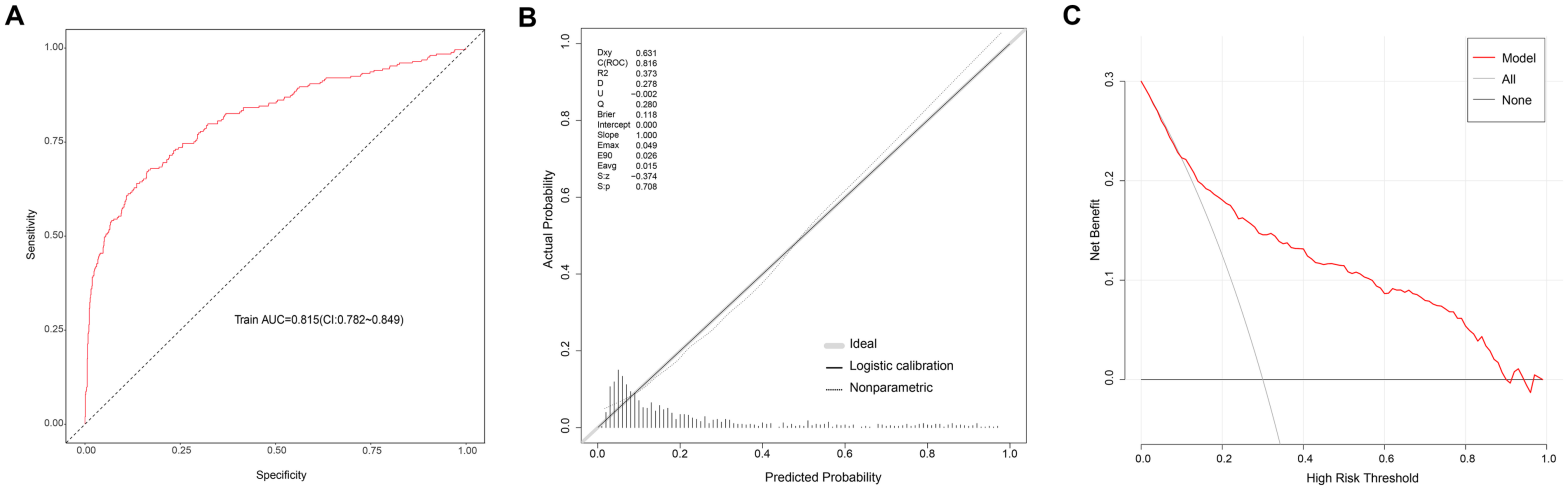
Evaluation of logistic model in the modeling group. **(A)** ROC curves. **(B)** Calibration curves. **(C)** Decision-curve analysis.

**Figure 4:**
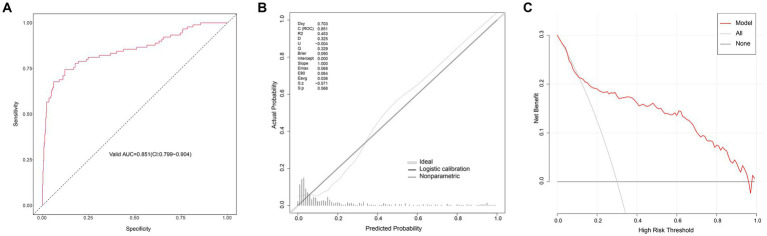
Evaluation of logistic model in the validation group. **(A)** ROC curves. **(B)** Calibration curves. **(C)** Decision-curve analysis.

The AUC of the logistic regression model in the modeling group was 0.815 (95CI: 0.782–0.849) (Figure 3A), showing good discrimination ability; the *p* value of the calibration chart was 0.708, with Brier scaled score of 0.118, calibration slope of 1.000, and R2 of 0.373, indicating the good fit (Figure 3B). The DCA curve was far away from the two extreme curves, indicating its good clinical applicability (Figure 3C). The AUC in the validation group was 0.851 (95CI: 0.799–0.904) (Figure 4A); the *p* value of the calibration chart was 0.568, with Brier scaled score of 0.090, calibration slope of 1.000, R2 of 0.453 (Figure 4B); the DCA curve was far away from the two extreme curves (Figure 4C), suggesting that the prediction model performed well in discrimination, goodness of fit and clinical applicability in the validation group.

### 3.4 Comparison with machine learning models

**Figure 5:**
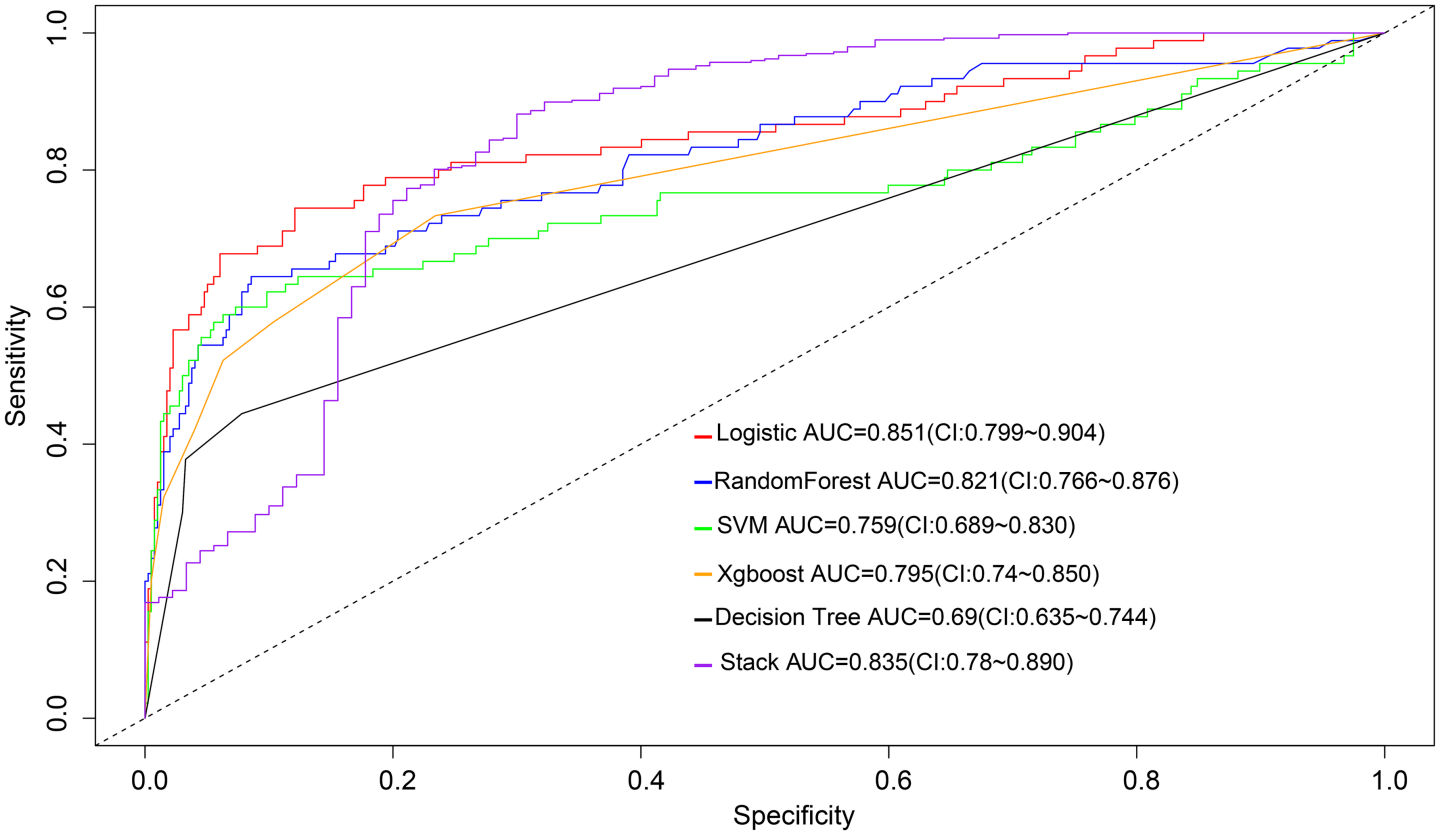
ROC curves for the logistic model and five machine learning models to predict poor treatment risk. SVM, Support Vector Machine; Xgboost, extreme gradient boosting.

The AUC values of machine learning models in the validation group were as follows: random forest (0.821), SVM (0.759), Xgboost (0.795), decision tree (0.690), and integrated machine learning model (0.835) (Figure 5). The discriminative ability of the logistic regression model was significantly higher than that of the models established by SVM, Xgboost and decision tree, but equivalent to that of the models established by random forest and integrated models (Supplementary Table S3).

## 4 Discussion

In this study, a clinical prediction model for poor treatment outcomes in the intensive phase in patients with initial pulmonary TB treatment was established. There were five clinical indicators enrolled in this model, including the presence of diabetes, lung cavities, TBTB, declined hemoglobin, and increased CRP. The model established in this study performs well in terms of discrimination, calibration and clinical applicability. This nomogram prediction model could be used as an effective tool for predicting and screening the treatment outcomes in the intensive phase.

The model developed in this study shows that the presence of diabetes in pulmonary TB inpatients is a risk factor for the poor treatment outcomes in the intensive phase. Diabetes mellitus is an important risk factor for TB, which can increase TB incidence and affect patient treatment response (19). Studies have shown that diabetes is associated with an increased risk of adverse outcomes in pulmonary TB. Therefore, there is an urgent need to screen for diabetes in TB patients and to implement interventions to improve the outcomes of pulmonary TB patients combined with diabetes (20–22). Intrapulmonary lesion resorption is better in TB patients with effective glycemic control compared with that in patients without effective glycemic control (23). The presence of lung cavities is an important factor for poor prognosis, diseases recurrence, and drug resistance development in pulmonary TB patients. The presence of lung cavities can hinder the penetration of anti-TB drugs into the lesions due to poor vascularization and necrotic tissue, thereby reducing treatment effectiveness (24, 25). The present study indicated that the presence of pulmonary cavities in baseline information before anti-TB treatment was a risk factor of poor treatment outcomes, which is consistent with previous studies (21, 26). Poor glycemic control further exacerbates the immune dysfunction of pulmonary TB patients and makes them more susceptible to lung cavities, which, in turn, increases the risk of treatment failure (27, 28).

TB is a chronic consumptive infectious disease, which will lead to the decrease of hemoglobin level and anemia. A multicenter cohort study showed that the frequency of adverse outcomes of pulmonary TB treatment increased with the severity of anemia (29). Although hemoglobin levels increase with the success of anti-TB treatment, the clinical recovery of anemic TB patients is slower during the intensive phase of treatment than that of non-anemic patients (30). The present research proved that the decrease in hemoglobin level can be a powerful predictor for treatment failure in the intensive phase of TB patients, and the severity of anemia was proportional to the risk of treatment failure. In addition, a feature of pulmonary TB is systemic inflammation. Previous studies have discovered that CRP can be used as a biomarker for evaluating the severity and treatment effect of TB (31, 32). The change of CRP may assist in evaluating the response of anti-TB treatment in the early stage, and identifying patients with increased risk of adverse outcomes. Compared with that of cured patients, the baseline CRP level in patients with failed outcomes is significantly higher (33, 34). This study showed that elevated CRP is an important predictor for poor treatment outcomes of pulmonary TB patients in the intensive phase.

It is reported that about 10–40% of pulmonary TB patients suffer from TBTB, and TBTB lesions often damage the tracheobronchial wall, resulting in its necrosis and tracheobronchial stenosis. Long-term bronchial stenosis, twisting and deformation can trigger local ventilation and blood flow dysfunction, which may lead to intractable TB lowering the effectiveness of the treatment, and even cause death (35, 36). The presence of TBTB in patients was hereby shown as a risk factor for poor treatment outcomes in the intensive phase. Early diagnosis of TBTB through radiological imaging and bronchoscopy, timely anti-TB treatment and interventional treatment under bronchoscope can reduce the risk of further aggravation of bronchial stenosis, and preserve the pulmonary ventilation function as much as possible to improve the prognosis (37).

A previous study indicated that machine learning models could accurately predict the treatment outcome of pulmonary TB patients (14, 16, 38). Therefore, several machine learning models were also hereby established to compare its effectiveness with the logical regression model in this study. The results suggested that the discriminative ability of logistic regression model was significantly higher than that of SVM, Xgboost and decision tree models. However, the efficiency was comparable to that of random forest and integrated models. Nevertheless, machine learning models often face challenges in clinical interpretation, making them difficult to popularize and apply in actual clinical practice (15, 39). The discrimination ability of the developed logistic regression model is not inferior to that of the machine learning model, but it is easier to interpret clinically. Therefore, this model can be better popularized and applied in the clinic.

Limitations of this study: (1) The data included in this study were from a single center. The patients from other regions might have different clinical features, resulting into different enrolled variables in the prediction model and varied prediction efficiency. Therefore, multi-center studies were required to validate our findings in the future. (2) Some patients were not included in this study due to information loss, which might bring a bias in patient population.

## 5 Conclusion

In this study, a prediction model was established to evaluate the risk of poor treatment outcomes in pulmonary tuberculosis patients during the intensive phase of treatment. The model performed well and can assist clinicians in implementing more targeted interventions to improve treatment success rates in pulmonary tuberculosis patients.

## Data Availability

The original contributions presented in the study are included in the article/Supplementary material, further inquiries can be directed to the corresponding authors.
