# Supplementary material for: Development of a clinical prediction model for poor treatment outcomes in the intensive phase in patients with initial treatment of pulmonary tuberculosis
Source: Front Med (Lausanne). 2025 Mar 26;12:1472295. doi: 10.3389/fmed.2025.1472295 (PMC11978639; doi:10.3389/fmed.2025.1472295)
Supplement: Supplementary file 1 [file Table_1.DOCX]

Table S1 The linear relationship analysis between continuous variables and logit(p).

| Variables | MLE of lambda | Score Statistic(z) | Pr(>\|z\|) |
| --- | --- | --- | --- |
| C-reactive protein (mg/L) | 1 | 1.8748 | 0.06082 |
| White blood cells (10*9/L) | 1 | -1.1974 | 0.23116 |
| Hemoglobin(g/L) | 1 | -0.4085 | 0.68292 |
| Platelet (10*9/L) | 1 | -0.1593 | 0.87345 |
| Albumin (g/L) | 1 | 0.9717 | 0.33120 |
| HDL (mmol/L) | 1 | 0.5697 | 0.56885 |

**Abbreviations:**HDL, High-density lipoprotein cholesterol.

Table S2. Variance inflation factor of variables.

| Variables | VIF |
| --- | --- |
| C-reactive protein (mg/L) | 1.908 |
| White blood cells (10*9/L) | 1.232 |
| Hemoglobin(g/L) | 1.366 |
| Platelet (10*9/L) | 1.144 |
| Albumin (g/L) | 2.077 |
| HDL (mmol/L) | 1.231 |
| Diabetes | 1.157 |
| Tumor | 1.025 |
| Pulmonary cavity | 1.234 |
| TBTB | 1.068 |

**Abbreviations:**HDL, High-density lipoprotein cholesterol; TBTB, Tracheobronchial tuberculosis.

Table S3 The AUCs comparison of models based on logistic regression, Randomforest, Decision tree, SVM, Xgboost and Ensemble.

| Model | AUC (95% CI) | P^a^ |
| --- | --- | --- |
| Logistic regression | 0.851 (0.799-0.904) | Reference |
| Randomforest | 0.821 (0.766-0.876) | 0.070 |
| Decision tree | 0.690 (0.635-0.744) | <0.001 |
| SVM | 0.759 (0.689-0.830) | 0.007 |
| Xgboost | 0.795 (0.740-0.850) | 0.007 |
| Ensemble | 0.835 (0.780-0.890) | 0.200 |

^a^ The significance was obtained by comparison to logistic regression model.
